# Supplementary material for: Clinical Study of Aspirin and Zileuton on Biomarkers of Tobacco-Related Carcinogenesis in Current Smokers
Source: Cancers (Basel). 2022 Jun 11;14(12):2893. doi: 10.3390/cancers14122893 (PMC9221101; doi:10.3390/cancers14122893)
Supplement: Supplementary file 1 [file cancers-14-02893-s001.zip › cancers-1713958-supplementary.pdf]

Supplementary Materials

Supplementary Figure S1: PGEM levels baseline, end-of-intervention and post-intervention

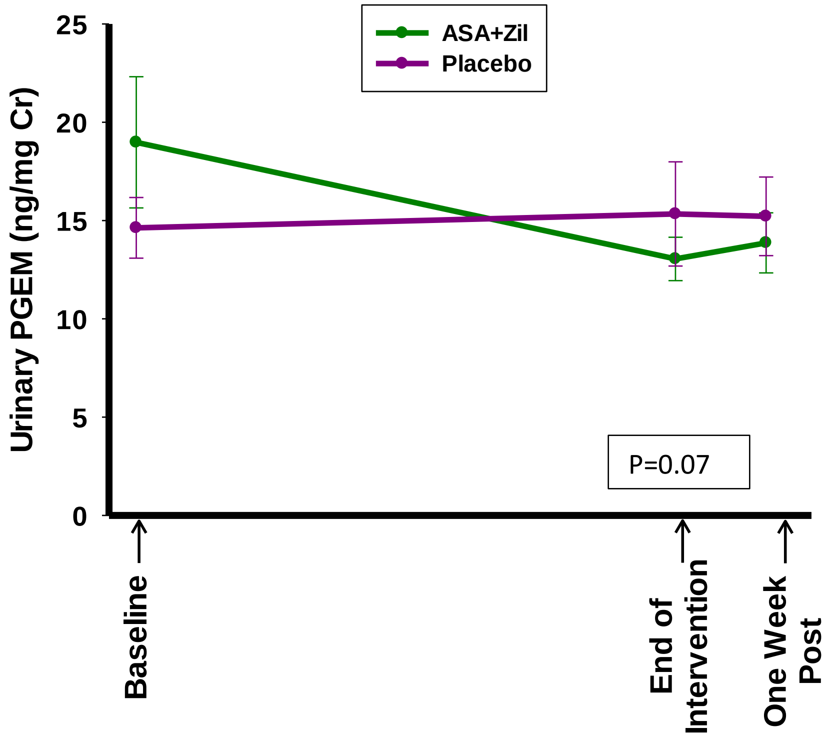

Supplementary Figure S2. Boxplots of the gene signature scores in the datasets used to derive the gene signatures. (**A-G**) Boxplots of the signature scores for each gene signature of interest detailed in the Supplementary Methods in the datasets in which the gene signature was derived.

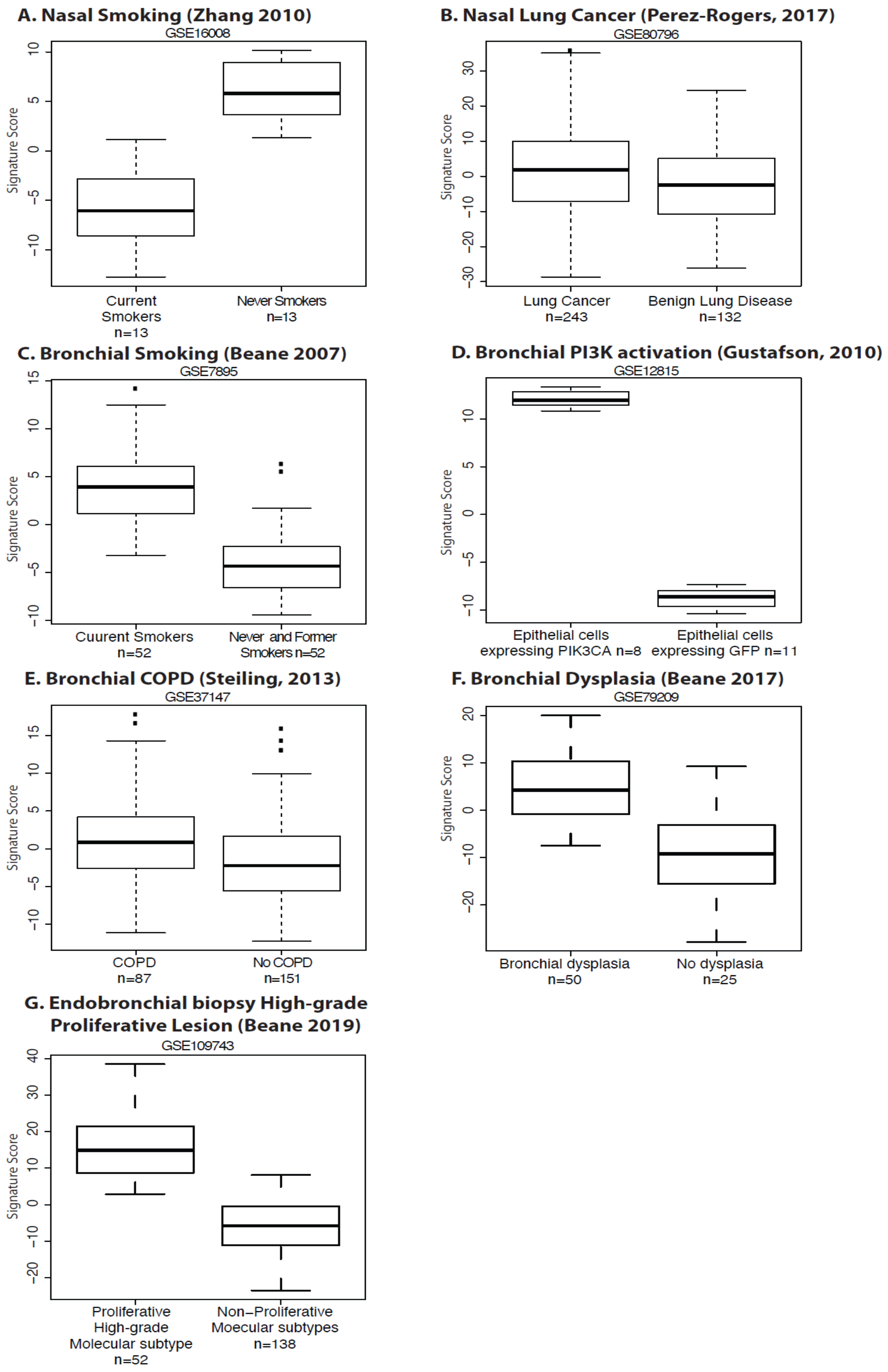

Supplementary Figure S3. Methodology used to score the aspirin plus zileuton samples for gene expression signature activation. We tested whether or not gene expression signatures associated with smoking, lung cancer, and COPD were modulated in the nasal epithelium by aspirin plus zileuton treatment. The gene expression signatures and the datasets in which each signature was derived is detailed in the Supplementary Methods. For each previously published gene expression signature, the corresponding gene expression data was downloaded from the NCBI Gene Expression Omnibus (GEO) using the study accession. For each gene signature dataset, ComBat was used to remove batch effects between the dataset (red) and the aspirin plus zileuton dataset (blue). ComBat adjusted gene expression values were z-score normalized across the combined aspirin and signature datasets. For each gene signature, the corresponding signature dataset was subset to include only the genes from that signature, and principal component analysis was conducted across the signature dataset. The first principal component (PC1) from each signature was adjusted to ensure the sign of each gene matched its direction in the signature in order to obtain a single score from both up- and down-regulated genes. The adjusted PC1 was projected into the aspirin and zileuton data to generate gene signature scores. Case 1 demonstrates an example where the gene signature is concordant (correlated or anti-correlated) with aspirin and zileuton-associated gene expression alterations.

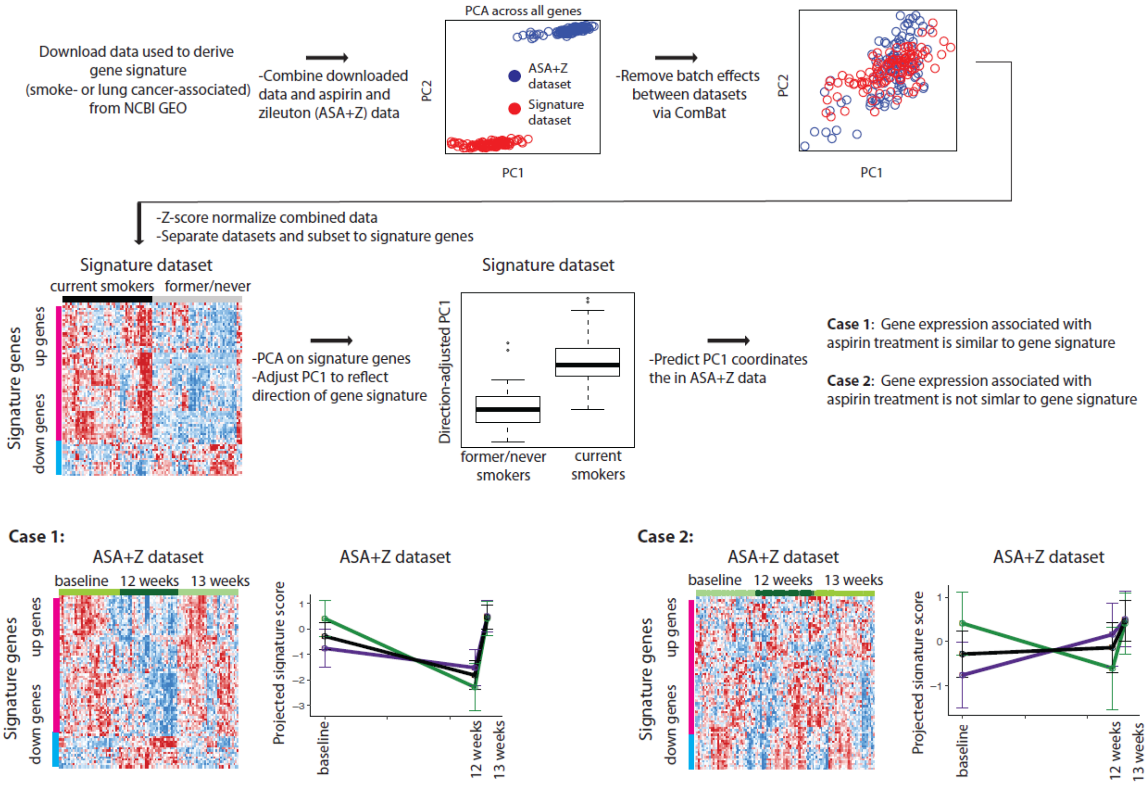

Supplementary Methods

Gene expression datasets used in the analysis

All of the below processed datasets were downloaded from the Gene Expression Omnibus (GEO).

Nasal smoking, GSE16008 (Zhang et al.2008): Paired bronchial and nasal epithelial cells were collected from healthy never (n=14) and current (n=13) smoker volunteers and profiled using Affymetrix Human Exon 1.0 ST Genechips (1). Zhang et al. reports a 119-gene signature that is associated with smoking in both the bronchial and nasal epithelium. In this study, the high-quality bronchial epithelial cell gene expression data (n=13 never and n=13 current smokers) and the 119-gene signature (107 genes after mapping to gene symbols, 62 up-regulated in current smokers) was used to score the samples in the ASA + zileuton data.

Nasal lung cancer, GSE80796 (AEGIS Study Team, 2017): Nasal epithelial brushings were prospectively collected from current and former smokers with pulmonary lesions suspicious for lung cancer in the Airway Epithelial Gene Expression in the Diagnosis of Lung Cancer (AEGIS) clinical trials (n=375 from AEGIS-1 and n=130 from AEGIS-2) and gene expression profiled using Affymetrix Gene 1.0 ST microarrays. Perez-Rogers et al. (2017) report a 535-gene signature associated with lung cancer derived using the AEGIS-1 samples (2). In this study, the nasal epithelial cell gene expression data (n=375 from AEGIS-1, n=243 with lung cancer and n=132 with benign disease) and the 535-gene signature (425 genes after mapping to gene symbols, 20 up-regulated in subjects with cancer) was used to score the samples in the ASA + zileuton data.

Bronchial smoking, GSE7895 (Beane et al.,2007): Bronchial epithelial cells were collected via bronchoscopy from disease-free never (n=21), former (n=31), and current smokers (n=52). RNA was isolated from each sample and gene expression was profiled using Affymetrix Human Genome U133A arrays. Beane et al. identified a 139 genes that were associated with smoking and were rapidly reversible upon smoking cessation(3). In this study, the bronchial epithelial cells from current smokers (n=52) and former/never smokers (n=52) and the 139-gene signature (81 genes after mapping to gene symbols, 66 up-regulated in current smokers) was used to score the samples in the ASA + zileuton data.

Bronchial PI3K activation, GSE12815 (Gustafson et al.,2010): As detailed in Gustafson *et al.*(4), recombinant adenoviruses were used to express the p110α isoform of PI3K in a quiescent mammary epithelial cells(4). RNA was collected eighteen hours post-infection (with PI3K isoform or GFP control) from multiple independent infections and profiled on the Affymetrix HT Human Genome U133A array. Gustafson et al. observed activation of the PI3K gene signature in the bronchial airways of smokers with lung cancer and smokers with dysplastic lesions. In this study, the 183-gene PI3K signature (133 genes after mapping to gene symbols, 87 up-regulated in PI3K over-expression) as well as the 8 samples over-expressing PI3K and the 11 GFP control samples were used to score the samples in the ASA + zileuton data.

Bronchial COPD, GSE37147 (Steiling et al., 2013): Bronchial epithelial cells were collected via bronchoscopy from current and former smokers with (n=87) and without (n=151) chronic obstructive pulmonary disease (COPD). RNA was isolated from each sample and gene expression was profiled using Affymetrix Human Gene 1.0 ST arrays. Steiling et al. identified a

98-gene signature associated with COPD status, FEV1 (forced expiratory volume in 1 second) percent, and FEV1/FVC (forced vital capacity) ratio. In this study, the 238 subjects with and without COPD and the 98-gene signature (88 genes after mapping to gene symbols, 50 up-regulated in COPD) were used to score the samples in the ASA + zileuton data.

Bronchial squamous dysplasia, GSE79209, GSE79210 (Beane et al, 2017). Bronchial airway epithelial cells were collected via bronchoscopy from smokers with and without premalignant lesions (GSE79209) and from high risk subjects via multiple bronchoscopy procedures to follow bronchial premalignant lesions as part of lung cancer screening (GSE79210). Bronchial brushes of normal appearing epithelium from 84 subjects (1 brush/subject) with and without PMLs were selected to undergo mRNA sequencing (mRNA-Seq) while ensuring balanced clinical covariates. Fifty-one bronchial brushes of normal appearing epithelium from 23 subjects were also profiled by mRNA-Seq (18 subjects had 2 procedures, and 5 subjects had 3 procedures) and utilized as a secondary biomarker validation set. In this study, a 280 gene signature was used to score the samples in the ASA + zileuton study.

Bronchial proliferative molecular subtype, GSE109743 (Beane et al, 2019). Bronchial epithelial cells were collected via bronchoscopy using RNA-Seq airway brushing and biopsies (divided into two cohorts: discovery and validation) obtained from high-risk smokers undergoing lung cancer screening via serial auto-fluorescence bronchoscopy procedures. mRNA sequencing was performed on a discovery cohort (DC) of samples comprising of endobronchial biopsies and brushes 2012 (n=30 subjects, n=197 biopsies, and n=91 brushings). mRNA sequencing was subsequently performed on a validation cohort (VC) of samples comprising of endobronchial biopsies and brushes (n=20 subjects, n=111 biopsies, and n=49 brushings). Four distinct

molecular subtypes (Proliferative, Inflammatory, Secretory, and Normal) were identified in the bronchial biopsies that correspond to a spectrum of biological and morphological alterations. In this study, a 200 gene signature was used to score the samples in the ASA + zileuton data.

Scores generated based on the bronchial gene expression-based lung cancer classifier

(Whitney *et al.,2015*) Bronchial epithelial cells were collected from current and former smoking subjects with (n=76) and without lung cancer (n=223) undergoing bronchoscopy for suspicion of lung cancer in a prospective, multi-center study. RNA was isolated from each sample and gene expression was profiled using Affymetrix Human Gene 1.0 ST arrays. A lung cancer diagnostic classifier was built based on a combination of 17 cancer-associated genes and gene expression predictors of smoking status, smoking history, and gender (n=6 additional genes), plus patient age(6). The 23-gene classifier was developed into a lung cancer diagnostic test known as PERCEPTA^{TM}, and was validated in an independent cohort (Silvestri et al, 2015) (GSE66499). The commercialized biomarker algorithm was used to score the samples in the ASA + zileuton data

References

1. Zhang X, Sebastiani P, Liu G, Schembri F, Zhang X, Dumas Y-M, et al. Similarities and differences between smoking-related gene expression in nasal and bronchial epithelium. Physiological Genomics. 2010;41:1–8.

2. AEGIS Study Team. Shared Gene Expression Alterations in Nasal and Bronchial Epithelium for Lung Cancer Detection. J Natl Cancer Inst. 2017;109.

3. Beane J, Sebastiani P, Liu G, Brody JS, Lenburg ME, Spira A. Reversible and permanent effects of tobacco smoke exposure on airway epithelial gene expression. Genome Biol. 2007;8:R201.

4. Gustafson AM, Soldi R, Anderlind C, Scholand MB, Qian J, Zhang X, et al. Airway PI3K pathway activation is an early and reversible event in lung cancer development. Sci Transl Med. 2010;2:26ra25.

5. Steiling K, van den Berge M, Hijazi K, Florido R, Campbell J, Liu G, Xiao J, Zhang X, Duclos G, Drizik E, Si H, Perdomo C, Dumont C, Coxson HO, Alekseyev YO, Sin D, Pare P, Hogg JC, McWilliams A, Hiemstra PS, Sterk PJ, Timens W, Chang JT, Sebastiani P, O'Connor GT, Bild AH, Postma DS, Lam S, Spira A, Lenburg ME. A dynamic bronchial airway gene expression signature of chronic obstructive pulmonary disease and lung function impairment. Am J Respir Crit Care Med. 2013 May 1;187(9):933-42.

6. Beane J, Mazzilli SA, Tassinari AM, Liu G, Zhang X, Liu H, et al. Detecting the presence and progression of premalignant lung lesions via airway gene expression. Clin Cancer Res 2017;23:5091-100.

7. Beane JE, Mazzilli SA, Campbell JD, et al. Molecular subtyping reveals immune alterations associated with progression of bronchial premalignant lesions. *Nat Commun*. 2019;10(1):1856. Published 2019 Apr 23. doi:10.1038/s41467-019-09834-2.

8. Whitney DH, Elashoff MR, Porta Smith K, Gower AC, Vachani A, Ferguson JS, et al. Derivation of a bronchial genomic classifier for lung cancer in a prospective study of patients undergoing diagnostic bronchoscopy. BMC Med Genomics. 2015;8:18.

9. Silvestri GA, Vachani A, Whitney D, Elashoff M, Porta Smith K, Ferguson JS, et al. A Bronchial Genomic Classifier for the Diagnostic Evaluation of Lung Cancer. N Engl J Med. 2015.

Supplementary Tables

Supplementary Table S1: Baseline characteristics for participant cohort with gene scores

Variable	All (n=40)	ASA+Zileuton (n=19)	Placebo (n=21)	P[c]
Age	52.90±9.07[a]	49.74±6.83	55.76±10.01	**0.03**
BMI	28.29±7.76	29.09±7.46	27.55±8.13	0.54
Packyears	35.15±11.60	32.37±9.55	37.67±12.89	0.15
Male	21 (52.50%)[b]	11 (57.89%)	10 (47.62%)	0.55
White	33 (82.50%)	17 (89.47%)	16 (76.19%)	0.66
Hispanic	6 (15.00%)	2 (10.53%)	4 (19.05%)	0.66

[a]mean±standard deviation
[b]frequency (%)
[c]derived from two-sample t test for continuous variables and Fisher's exact tests for categorical variables

Supplementary Table S2: Summary of baseline and changes in the gene-expression signature scores by treatment group

Score	All (n=40)	ASA+Zileuton (n=19)	Placebo (n=21)	P[b]
Beane (32)				
V2	-1.01±6.87	-2.11±7.10	-0.02±6.67	0.34
V5	-0.56±7.14	-1.38±7.54	0.19±6.87	
V6	1.94±7.52	1.02±8.80	2.77±6.27	
V5-V2	0.46±5.50	0.74±5.48	0.20±5.64	0.76
V6-V2	2.89±6.60	2.60±6.83	3.16±6.54	0.80
Steiling (21)				
V2	-0.08±2.66	-0.02±2.70	-0.13±2.68	0.90
V5	-0.43±2.65	-0.52±3.05	-0.36±2.30	
V6	0.64±2.53	-0.03±2.55	1.24±2.43	
V5-V2	-0.35±2.28	-0.49±2.22	-0.23±2.38	0.72
V6-V2	0.58±2.48	-0.21±1.88	1.29±2.77	0.06
Beane (29)				
V2	0.17±3.32	0.32±3.33	0.04±3.38	0.80
V5	-0.53±3.38	-0.68±3.50	-0.39±3.34	
V6	0.19±3.04	-0.50±2.95	0.81±3.05	
V5-V2	-0.70±3.12	-1.00±2.87	-0.43±3.37	0.57
V6-V2	-0.19±3.42	-1.37±2.69	0.88±3.71	**0.04**
Perez - Rogers (28)				
V2	-0.80±11.32	-0.64±10.10	-0.94±12.56	0.93
V5	1.20±10.64	1.18±8.85	1.22±12.26	
V6	-1.00±9.50	1.34±8.55	-3.09±10.03	
V5-V2	2.00±9.94	1.82±8.63	2.17±11.21	0.91
V6-V2	0.28±9.65	2.78±6.35	-1.98±11.58	0.12
Perez-Rogers Small (28)				
V2	-0.24±3.21	0.02±2.84	-0.47±3.57	0.64
V5	0.05±3.15	0.29±2.64	-0.17±3.60	
V6	0.02±2.69	1.01±2.52	-0.88±2.56	
V5-V2	0.29±3.13	0.28±2.98	0.30±3.32	0.98
V6-V2	0.36±3.04	1.10±2.22	-0.30±3.55	0.16
Gustafson (31)				
V2	-0.10±1.69	-0.16±1.55	-0.04±1.84	0.83
V5	-0.03±1.61	-0.47±1.76	0.38±1.37	
V6	0.21±1.80	-0.25±1.35	0.63±2.07	
V5-V2	0.07±1.65	-0.32±1.57	0.42±1.68	0.16
V6-V2	0.30±2.06	-0.16±1.63	0.71±2.35	0.19
Whitney (30)				
V2	0.70±0.15	0.76±0.10	0.65±0.17	**0.01**
V5	0.73±0.15	0.68±0.18	0.78±0.09	
V6	0.62±0.19	0.66±0.18	0.58±0.20	
V5-V2	0.03±0.23	-0.08±0.21	0.13±0.20	**<0.01**[c]
V6-V2	-0.08±0.21	-0.09±0.17	-0.07±0.25	0.71

[a]mean±standard deviation
[b]derived from two-sample t test
[c] after adjusting for the V2 (baseline) value, the p-value becomes 0.06>0.05 for V5-V2. This indicates the difference in V5-V2 probably is due to the significant difference at baseline.

Supplementary Tables S3-S9: See Excel File

Table Legends:

Table S3. Gene signature scores for each sample in the ASA plus zileuton trial.
Table S4. Genes associated with ASA plus zileuton treatment. The gene symbol, Entrez Gene probe ID, t-statistic, p-value, and the direction of change are reported.
Table S5. Genes associated with one-week post- ASA plus zileuton treatment. The gene symbol, Entrez Gene probe ID, t-statistic, p-value, and the direction of change are reported.
Table S6. Hallmark pathways enriched (FDR<0.25) among genes up-regulated with ASA plus zileuton treatment by Gene Set Enrichment Analysis. The pathway name, size of the pathway, enrichment score (ES), normalized enrichment score (NES), Nominal p-value, FDR, and FWER p-value are reported.
Table S7. Hallmark pathways enriched (FDR<0.25) among genes down-regulated with ASA plus zileuton treatment by Gene Set Enrichment Analysis. The pathway name, size of the pathway, enrichment score (ES), normalized enrichment score (NES), Nominal p-value, FDR, and FWER p-value are reported.
Table S8. Hallmark pathways enriched (FDR<0.25) among genes up-regulated one-week post-ASA plus zileuton treatment by Gene Set Enrichment Analysis. The pathway name, size of the pathway, enrichment score (ES), normalized enrichment score (NES), Nominal p-value, FDR, and FWER p-value are reported.
Table S9. Hallmark pathways enriched (FDR<0.25) among genes down-regulated one-week post- ASA plus zileuton treatment by Gene Set Enrichment Analysis. The pathway name, size of the pathway, enrichment score (ES), normalized enrichment score (NES), Nominal p-value, FDR, and FWER p-value are reported.
